# Agenda Setting for Health Promotion: Exploring an Adapted Model for the Social Media Era

**DOI:** 10.2196/publichealth.5014

**Published:** 2015-11-25

**Authors:** Yousef Albalawi, Jane Sixsmith

**Affiliations:** ^1^ Health Promotion Research Centre National University of Ireland Galway Galway Ireland; ^2^ Public Health Administration Ministry of Health Medina Saudi Arabia

**Keywords:** agenda setting, health promotion, social media, Twitter, health communication, Saudi Arabia, road traffic accidents

## Abstract

**Background:**

The foundation of best practice in health promotion is a robust theoretical base that informs design, implementation, and evaluation of interventions that promote the public’s health. This study provides a novel contribution to health promotion through the adaptation of the agenda-setting approach in response to the contribution of social media. This exploration and proposed adaptation is derived from a study that examined the effectiveness of Twitter in influencing agenda setting among users in relation to road traffic accidents in Saudi Arabia.

**Objective:**

The proposed adaptations to the agenda-setting model to be explored reflect two levels of engagement: agenda setting within the social media sphere and the position of social media within classic agenda setting. This exploratory research aims to assess the veracity of the proposed adaptations on the basis of the hypotheses developed to test these two levels of engagement.

**Methods:**

To validate the hypotheses, we collected and analyzed data from two primary sources: Twitter activities and Saudi national newspapers. Keyword mentions served as indicators of agenda promotion; for Twitter, interactions were used to measure the process of agenda setting within the platform. The Twitter final dataset comprised 59,046 tweets and 38,066 users who contributed by tweeting, replying, or retweeting. Variables were collected for each tweet and user. In addition, 518 keyword mentions were recorded from six popular Saudi national newspapers.

**Results:**

The results showed significant ratification of the study hypotheses at both levels of engagement that framed the proposed adaptions. The results indicate that social media facilitates the contribution of individuals in influencing agendas (individual users accounted for 76.29%, 67.79%, and 96.16% of retweet impressions, total impressions, and amplification multipliers, respectively), a component missing from traditional constructions of agenda-setting models. The influence of organizations on agenda setting is also highlighted (in the data of user interactions, organizational accounts registered 17% and 14.74% as source and target of interactions, respectively). In addition, 13 striking similarities showed the relationship between newspapers and Twitter on the mentions trends line.

**Conclusions:**

The effective use of social media platforms in health promotion intervention programs requires new strategies that consider the limitations of traditional communication channels. Conducting research is vital to establishing a strong basis for modifying, designing, and developing new health promotion strategies and approaches.

## Introduction

### Background

Communication is a core component of many effective health promotion interventions and change processes at individual and community levels [1]. In the social media age, the emergence of eHealth communication is expected to significantly enhance the efficacy of health promotion programs. The evolution of social media stimulated a shift of the communication equation from a top-down, expert-to-consumer approach to a nonhierarchical, dialog-based strategy. Consequently, communication has become an individual and community enabler in terms of achieving development goals, including health development [2]. Korda [3] indicated that an important characteristic of Web-based interventions is the sense of empowerment that it endows people and groups as they make decisions related to health; this feature is a positive influence on communities and individuals who are actively aiming for healthy behaviors and lifestyle changes. With these considerations in mind, we investigated the use of the agenda-setting function of health promotion interventions in the social media era. Specifically, we examined the effectiveness of Twitter as a social media platform in influencing agenda setting among users in relation to road traffic accidents in Saudi Arabia.

### Road Traffic Accidents

Globally, road traffic accidents result in 1.24 million deaths and 20 to 50 million injuries per year, many of which cause permanent disabilities [4]. In Saudi Arabia, the 544,000 yearly accidents cause 7153 fatalities and more than 39,000 injuries [5]. Eighty-one percent of deaths in Ministry of Health hospitals are the result of road traffic accidents [6]. The World Health Organization recommendations emphasize the consideration of road safety as a public health issue [7], with a focus on persuading policy and decision makers to place road traffic accidents on their agendas as a major problem and implement measures for improving related interventions.

Maximizing the effectiveness of social media for the promotion and protection of health necessitates intervention programs based on a thorough scientific understanding of how communication and media action theories and models are prioritized [8,9]. Agenda-setting theory has been examined within the sphere of social media and shows promise for the promotion of effective health practices [10,11,12].

According to Kaplan and Haenlein, social media is “a group of Internet-based applications that build on the ideological and technological foundations of Web 2.0, and that allow the creation and exchange of User Generated Content” [13]. This definition covers many types of social media including Twitter, Facebook, and Instagram. These platforms have powerful characteristics which make them effective channels for communication-based activities. An interesting development in recent years is the significant increase in the availability of social media; this growth is expected to continue [14].

The development of social media has been recognized as an opportunity for the promotion of the public’s health demonstrated through the concept of infodemiology, a term coined by Eysenbach [15]. Infodemiology is the melding of health informatics and epidemiology and has been defined as “the science of distribution and determinants of information in an electronic medium, specifically the Internet, or in a population, with the ultimate aim to inform public health and public policy” [16,17].

Infodemiology is based on the idea that the vast quantities of communication data generated by social media can be used for public health [16]. We live in a digital world where people communicate using Internet channels supported by highly advanced technologies. These communication channels are characterized by an ability to track activities and collect information and data about them. For example, social media platforms generate data that reflect people’s behaviors and record, in real time, large parts of their daily life, including their health status [16,18]. When suitable metrics and measures are applied, these data can provide valuable information that can inform policies, strategies, and decisions for public health at the level of policy makers and of the population [17].

These data provide a new level of information that was not measurable before this era [17]. Currently, only a small proportion will be analyzed (in 2013, only 5% of these data were analyzed [19]) due to a lack of methods and measures for collecting, analyzing, and interpreting such data [16,17,20]. Nevertheless, infodemiology advances our understanding and provides methods that can move public health to a new level of practice and research [16,17]. Applications of infodemiology can harmonize the research and practice of public health through the analysis of so-called “big data” in the era of social media [16,17]. Examples of infodemiology applications include tracking user activities on microblogging platforms such as Twitter [16]. This study explores user activities on Twitter in relation to public health and as such can be positioned in the context of infodemiology.

### Twitter

Twitter is “an information network made up of 140-character messages called Tweets” [21]. It is a social and microblogging service that enables participants to post messages and follow others’ posts. Outside China, 53% of the Internet population has Twitter accounts, and 69% of online adults browse Twitter [22]. The 2015 statistics for Saudi Arabia show that in a population of 28 million, more than 18 million are Internet users [23], 60% of whom have Twitter accounts and 33% of Internet users are active Twitter users [22]. Apart from being among the top-ranked countries in terms of registered users, Saudi Arabia is number one globally in terms of visitation rates (logged-out users) [22].

### Agenda-Setting Theory

**Figure 1:**
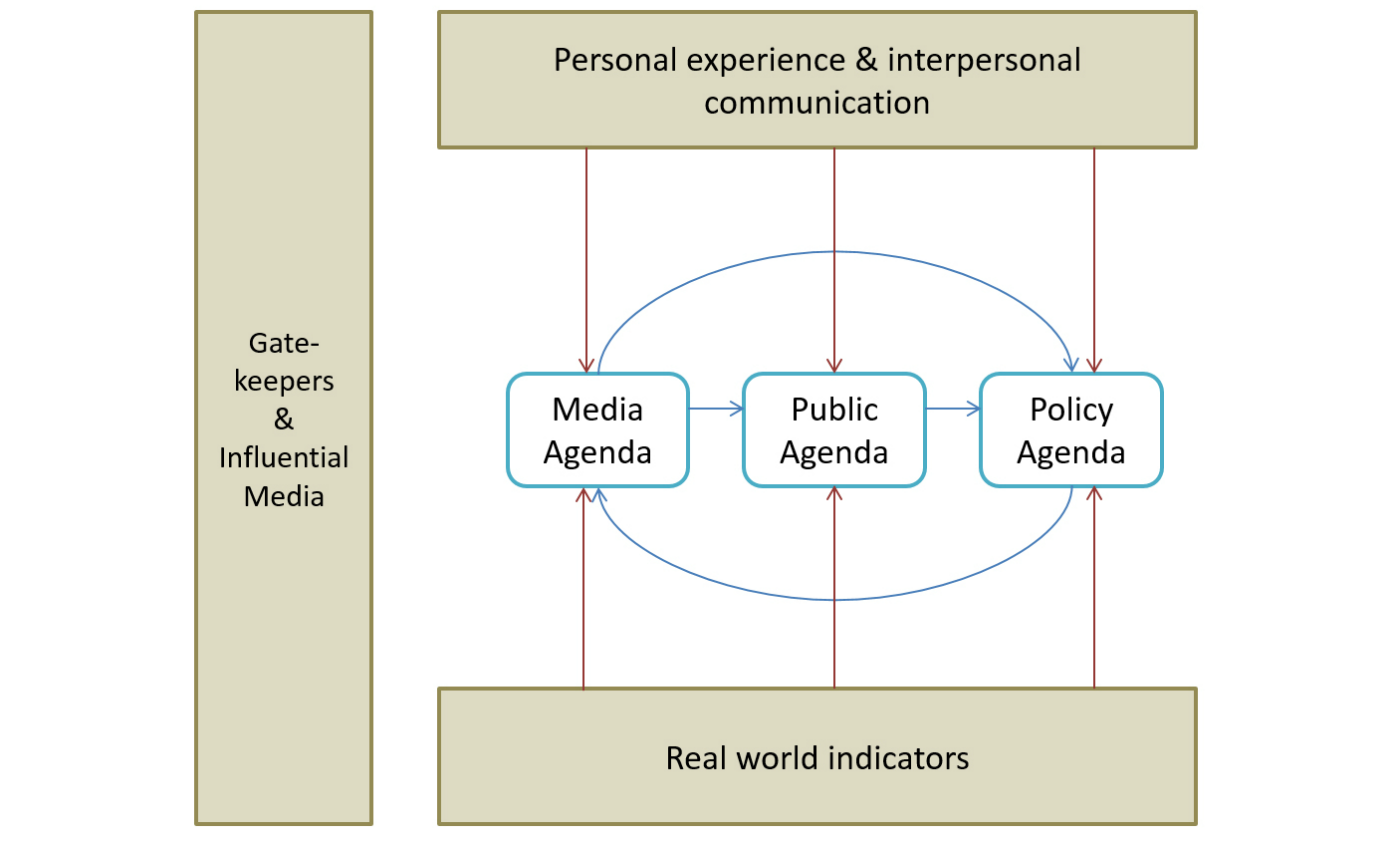
The three main components of the agenda-setting model.

Lippman [24] first expressed the idea of agenda setting, which was subsequently developed by Lasswell [25] and Cohen [26], culminating in agenda-setting theory through the work of McCombs and Shaw [27]. The core concept of agenda setting assumes that media stimulates the awareness of people regarding certain issues. This assumption is grounded on two main principles: (1) media shapes and filters reality before presenting it to people and (2) these channels determine the priority with which individuals regard salient issues [28]. Rogers and Dearing [29] proposed an agenda-setting model that comprises three components: media agenda, public agenda, and policy agenda. Each of these agendas represents issues that are the chief concerns of a particular stakeholder. The interrelationship among these components forms the core of agenda-setting theory [30]. Figure 1 shows the process of agenda setting among the three main components according to Rogers and Dearing’s model [29].

As indicated in the model, media agenda setting refers to traditional media organization decisions on which issues to discuss through their channels. Public agenda setting revolves around the issues that are considered important to the general public. Policy agenda setting involves official organizations or government agencies that determine which issues are important and worthy of discussion [31].

### Agenda Setting for Health Promotion

Kozel et al [32] developed agenda setting in the context of public health and health promotion through the process of health promotion agenda-setting [12,32,33,34]. Agenda setting is about the interrelationship of the domains of policy, media, and public agendas; health promotion agenda-setting is about how health issues move through agendas to the point that they become actionable by policymakers [35]. Health promotion agenda-setting shifts the focus from the traditional health education target of individual risk behavior change to the formulation and adoption of innovative health policies which advocate for the public’s health at population level [12,32]. Kozel et al [12,34], in response to an identified gap—the omission of agenda setting from health promotion planning models relating to innovation and diffusion—have developed a model of the health promotion agenda-setting process. The construction of this model includes the interrelated constructs of the media, policy, and public agendas with the integration of the seven responsibilities of health educators: assessment, planning, implementation, coordination, evaluation, acting as a resource person, and advocating for health [34]. Through the development of health promotion agenda-setting, including lessons learned from its practical application, a range of factors has emerged that enhances the diffusion of health promotion and disease prevention innovations [33]. These include characteristic factors such as demographic descriptors; design factors such as strategies and methods used; and mechanism factors such as shared vision, synchronicity, salience, and social justice [33]. Kozel et al [33] identify ten key activities for agenda setters to use in practice, two of which are tailoring strategies to prioritize a health issue in a population and sustaining salience of an issue in the domains of policy, media, and public agendas. The application of health promotion agenda-setting in practice enables a comprehensive, planned, innovative, and sustainable course of action which facilitates prioritization of public health problems and the identification of alternative solutions [12]. Health promotion agenda-setting contributes to health promotion leadership and provides a mechanism through which to improve the formulation and adoption of health policy.

In addition to the work by Kozel and colleagues on the development and application of health promotion agenda-setting, the concept and components of agenda setting have been used in public health and health promotion in a range of areas [36-42]. Understanding, researching, and implementing the use of agenda setting for health promotion practice will improve its performance and boost intervention outcomes [12]. This is particularly important in the era of social media, a relatively new addition to the media landscape that warrants further exploration in the context of agenda setting.

### Agenda Setting in the Social Media Era

Agenda-setting initiatives have been extensively studied and developed by researchers and practitioners. New frames and models have been proposed with emphasis placed on the ideal match between changes people and societies are undergoing in the social media era and agenda setting for public health [43]. Given that social media was nonexistent during the introduction and early development of agenda-setting theory, this has not been comprehensively investigated in previous research [44].

Simple application of agenda setting in the era of social media does not reflect the complex process of communication resulting from the use of social media platforms [44]. We argue that understanding agenda-setting theory in the social media era should cover two levels of engagement: the first centers on agenda setting within the social media sphere and the second is related to the position of social media within the classic agenda-setting process implemented in the real world. Here we propose social media in the agenda-setting context as an independent body governed by its own agenda..

### Social Media Agenda Setting

As proposed by Dearing and Rogers [45], agenda setting is best understood as a process of interaction; it therefore revolves around the flow of agendas from one component to another. Within the agenda-setting process, an important task is to identify who owns specific agendas and who interacts with other stakeholders.

#### Individual Agenda

Social media offers numerous platforms where people can communicate and interact. One of the most important changes in agenda setting within the social media realm is the shift in power towards the public in terms of control over communication; this shift was triggered by the fact that with social media technologies, individuals become active producers instead of functioning merely as receivers of information. Bekkers et al [46] argue that Web 2.0 has shifted political mobilization from a traditional mass-oriented movement to one driven by individuals and small groups of people.

Furthermore, individuals differ significantly in how they respond to the media agenda [47]. The power that individuals have gained in the social media era enables them to directly communicate their arguments, opinions, and agendas to the world. Supported by highly interactive features and user-generated content, social media platforms allow individuals to control what they receive, from whom, and how much according to their interests [48]. Tran and Johnson [49] claim that one of the opportunities provided by social media to agenda-setting research is the empowerment of individuals in developing their personal agendas [49]. We argue that such opportunity extends not only to development but also to influence over agendas. In real communities, an individual is a member of the public and thus can adopt and influence a given agenda advocated by a specific community. Similarly, an individual can hold membership in any organization and assist this organization by adopting and influencing its agenda. In classic agenda setting, individuals are always regarded as members of the public because influence is acquired through a process called “agenda melding” [39]. Although the emergence of social media does not cancel the role of agenda melding, it may extend the role of individuals by assigning them effective positions within the social process. This perspective is supported by the findings of Althaus and Tewksbury [50] and Conway and Patterson [51], who illustrated the differences in the power of individuals to control communication between traditional and social media.

#### Organizational Agenda

Social media has also redistributed the power to control communication at the organizational level. The nature of social media has allowed many types of organizations—not only media and policy institutions—to contribute to agenda setting. Similar to the shift in power at the public and individual level, changes at the organizational level have translated to organizational influence over and interaction with various agendas.

The effect of the presence of health organizations on these platforms has been explored in recent studies [52,53]. These studies include the examination of factors such as those associated with the organizations’ ability to engage and measures that directly affect the organizations’ influence [53].

The organizational agenda is not a new concept. Berger [31] pointed out that organizations are effective agenda-setting actors that can establish agenda through funding, lobbying, and advertising, thereby influencing the specific issues that are discussed in societies.

The authors propose to regard organizations as essential stakeholders in agenda setting because they can interact with different community actors, including the public, media, and policy makers.

### A New Contributor to the Agenda-Setting Process

As previously stated, the nature of social media with its two-way communication platforms and channels differs completely from that of traditional one-way mass communication channels. The social media age has driven changes in the manner by which information is disseminated. This era has decentralized traditional communication, thereby diminishing its power in shaping the issues that people think about [49]. Researchers have examined the relationship between traditional media (eg, newspapers and television) and social media (eg, Twitter and YouTube, a video-sharing website) [48,54]. The findings suggest that the social media realm is an independent arena that can affect and be affected by traditional media [54]. Research confirms traditional media’s influence over the social media agenda and vice versa [45,46,55].

McCombs [47] and Meraz [56], among others, have highlighted the manner by which social media influences agenda setting within the traditional media realm. An interesting finding is that the influence of social media not only covers the traditional media agenda but also extends to public and policy agendas [49]; these new channels affect the entire agenda-setting process. In exploring the relationship between social media and other agenda-setting components, many researchers distinguish social media from traditional media. An example is the separate examination of social media’s influence on public and policy agendas [57]. McCombs [47], who pointed out that social media redesigned agenda setting, supports this approach by adding a new contributor (ie, social media) to the process.

Collectively, the aforementioned studies focused not only on the discrepancy between traditional and social media in their effectiveness as communication tools but also on the independence of social media as an agenda-setting channel. Their findings suggest that studying social media as part of traditional media in the agenda-setting context is an unsuitable framework from which to understand the complexity of the agenda-setting process within the arena occupied by modern media innovations. About 70% of journal articles that explored agenda setting in the social media age are concentrated in intermedia agenda setting between new and traditional media [49]. Yet, the findings on social media as an independent channel [55] lend support to the claim that social media redistributed the power of agenda setting by adding a new domain to classic agenda-setting theory. We argue that social media can be regarded as a separate body within the agenda-setting process, as ideas from this perspective have been previously put forward in the literature. Meraz, for example, proposed social media as a new component of agenda setting, although he treated the new channels within as traditional media [56]. On these grounds, we propose a model for agenda setting in the social media era that reflects two levels of engagement: agenda setting within the social media sphere and the position of social media within classic agenda setting. The capability of the proposed model was assessed on the basis of the hypotheses formulated in this work.

### Study Hypotheses

**Figure 2:**
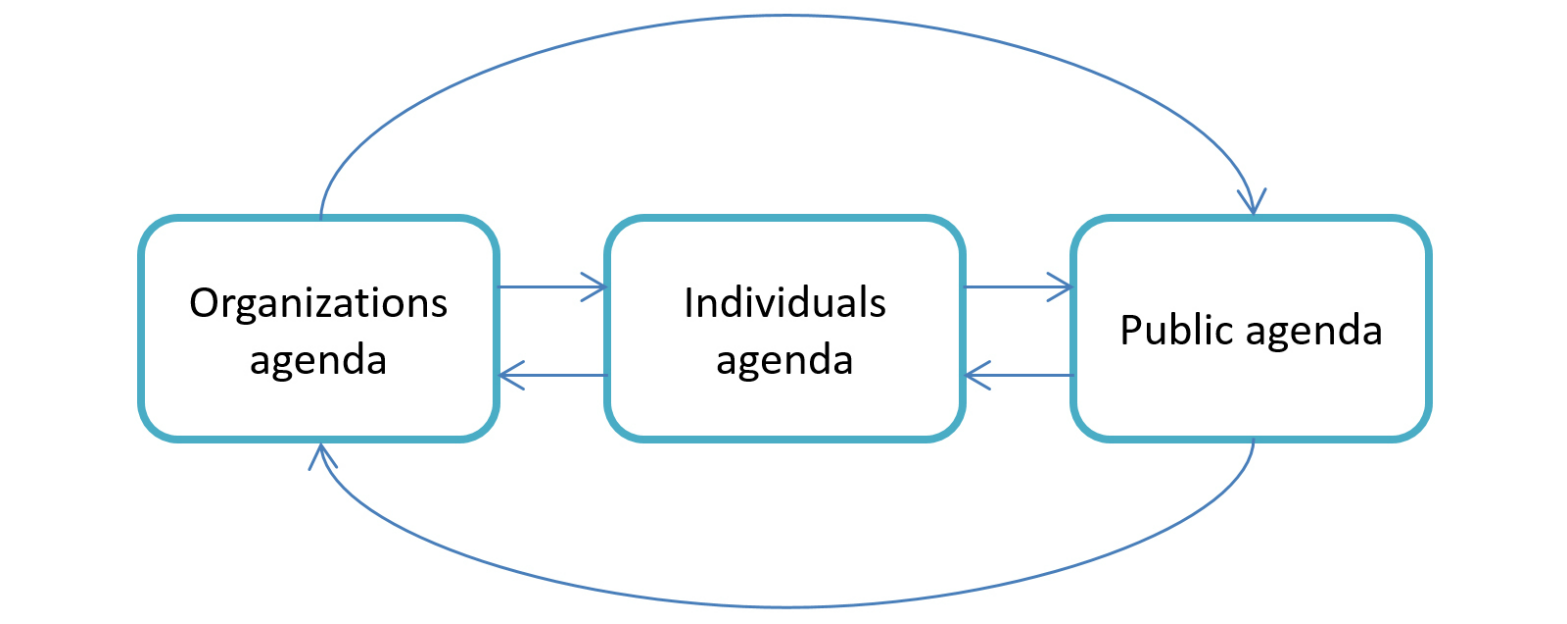
Adapted model of agenda setting within social media.

Hypothesis 1 (H1) revolves around the agenda-setting process within social media and suggests a new model fitted to the uniqueness of agenda setting under a social media interface. H1 maintains that individual and organizational agendas constitute a new body of plans and schemes instead of falling within the category of media and policy agendas (see Figure 2).

To validate H1, we put forward the following subhypotheses:

H1-1a: Individual accounts are the most dominant accounts.

H1-1b: Organizational accounts are more dominant than media and policy accounts.

We used three measures to determine the validity of H1-1a and H1-1b: rank scale measures for retweet impressions, total impressions, and total amplification multipliers (see Table 1 for definitions of terms). Data on contributor interactions were used as support measures. Two measures were adopted to differentiate between public and individual personal agendas: the average of total impressions and the amplification multiplier. The total impressions indicates the accumulated number of times a tweet was received, and the average is a measure of how a single account can be an influential factor in agenda setting compared with other account types. We therefore propose an additional subhypothosis:

H1-1c: Individual accounts represent individual personal agendas in addition to public agendas.

**Table 1:** Dataset variables.

Terminology	Definition
Retweets	Number of times a tweet is reposted or forwarded
Deliveries	Number of accounts to which a tweet is posted initially (equal to the number of followers the user has at that time)
Total impressions	Number of accounts that received the tweet; this includes direct post, retweets, and replies.
Retweet impressions	Number of impressions retweets of this tweet have generated
Amplification multiplier	The rate of amplification based on the tweet spread by retweets [(total exposure − impressions) / impressions] + 1

The data on contributor interactions were also used to determine the degree of influence of individual accounts as targets. We assume that when an account is targeted by other account types, these accounts represent individual agendas rather than public agendas. To examine nonaccount agendas, a critical requirement is determining that influence goes beyond accounts with special characteristics. For example, degree of influence is not restricted by a specific level of popularity. We thus propose H1-2:

H1-2: No correlation exists between account popularity and an account’s degree of influence; that is, tweets that are extensively disseminated can be created by accounts with only a few followers.

We used the correlation between two pairs of measures to test H1-2: the correlation between deliveries (number of times a tweet was received) and retweet impressions and that between impressions and the amplification multiplier.

**Figure 3:**
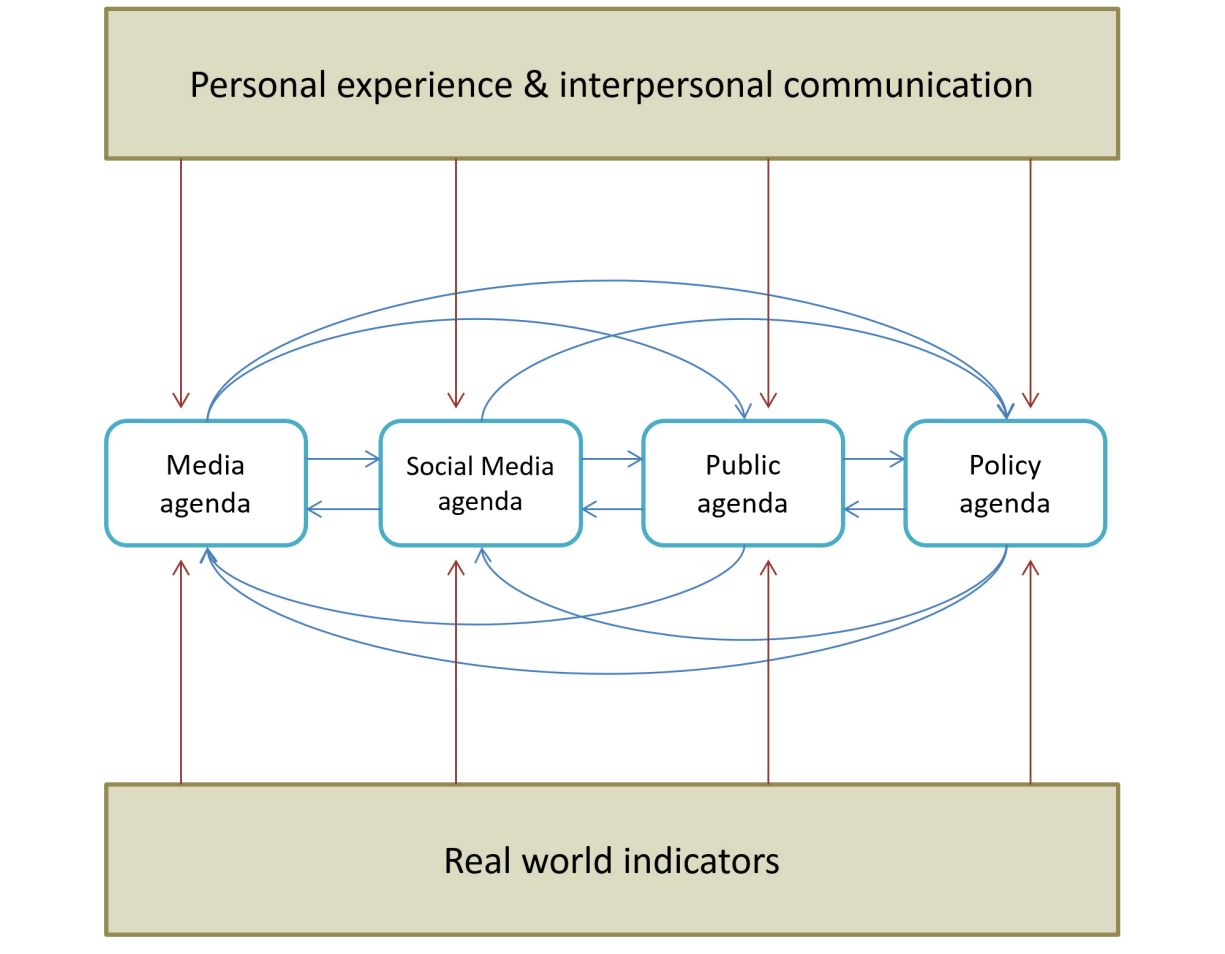
Proposed agenda setting model in the social media era.

Hypothesis 2 (H2) is related to the position of the social media agenda within classic agenda-setting theory. The theory posits that social media are new components incorporated into the three main elements of the classic agenda setting proposed by Rogers and Dearing [29] (Figure 3).

Determining the validity of H2 necessitates an investigation into the relationship between the social media agenda and the three other agenda types (media, public, and policy). However, the data collected in this study are useful only in exploring the relationship between Twitter as a social media platform and newspapers as traditional media channels. The collected data also lack many of the characteristics required from evidentiary sources (ie, a 90-day data collection period is a short time frame.). Despite these limitations, the data can provide valuable insights into the interaction between Twitter and newspaper agendas.

H2 is articulated thus:

H2: The trend of social media mentions is similar to that of newspaper mentions.

## Methods

### Design

This study is part of exploratory research aimed at testing the capability of traditional communication theories in understanding social media platforms related to health promotion practice.

Exploratory research is preliminary research that contributes to the formulation and identification of hypotheses that show some merit in being followed up by confirmatory research [58,59]. The formulation of this hypothesis is not usually restricted, and a more flexible approach is used [59,60]. This study suggests appropriate hypotheses that fit with the aim of the study overall. In addition, the statistical tests used to examine these hypotheses, the analysis procedures employed, and the intervention that developed as part of the study harmonize the spirit of exploratory research [59,60].

The study used an important concern of public health, that of road traffic accidents. Based on the conceptual frames [61,62] and the selected message design [63,64], tweets about road traffic accidents were developed, pilot tested, and approved by a university research ethics committee. The study disseminated the tweets through the Saudi Ministry of Health Twitter account.

Immediately after completion of this Twitter intervention, a national campaign on road safety was run through various traditional and social media channels. The campaign enhanced the dynamics of mentioning the keywords of the study. Such enhancement does not bias the study as it reflects the normal dynamic of interactions targeted by the study to be examined. Furthermore, the collected data covered the periods before and after the campaign. Textbox 1 presents examples of intervention tweets as well as other users’ tweets (see Multimedia Appendix 1 for the original Arabic texts).

**Examples of intervention tweets and tweets by other users that mention the keywords road traffic accident.**

Intervention tweets:

- Good health is a major resource for social, economic, and personal development and an important dimension of quality of life.
- 81% of all deaths in Ministry of Health hospitals are due to road traffic accidents.
- Road safety is a public health issue which involves health as well as other sectors that have the responsibility to be engaged in road traffic accident prevention.

Tweets by other users:

- Your life is a candle; do not extinguish it. Stats: it is estimated that the number of traffic accidents will reach 1 million in the next 8 years.
- Traffic accidents cost SR 1.9 trillion (US $518 billion) globally each year.
- Did you know that in Saudi Arabia one person every hour is killed in a traffic accident?!!

### Data Collection

Twitter data can be accessed directly from service profiles. Many third-party providers also offer Twitter statistics and analysis services. For instance, Tweetreach offers licensed access to the full Twitter “firehose” through Gnip, a licensed data reseller [65]. Increasing numbers of researchers are using these tools [66-72]. Account type was used as a variable for determining the most effective contributors to promoting road traffic accident agendas. Mentions of specific keywords as well as related variables (who tweeted messages, when messages were tweeted, to whom tweets were addressed, and how messages were tweeted) were the indicators used to measure contribution.

To validate the hypotheses, we collected data from two primary sources: Twitter activities and Saudi newspapers. As previously stated, keyword mentions served as indicators of agenda promotion; for Twitter, interactions were used to measure the process of agenda setting within the platform. Data from both sources were collected in a 90-day period from January 1 to March 31, 2014. Three Arabic keywords that are highly associated with road traffic accidents were considered in the analysis. The English translations of these keywords are “traffic accidents,” “the traffic accidents,” and “road accidents.” Tracking mentions of predefined keywords have been used in previous studies although for different research purposes [71,73,74].

In collecting the Twitter data, we used the Tweetreach service to collect all tweets that contained the keywords. Many researchers have used this tool. For example, it has been employed in examining the use of Twitter as a platform for sharing information about medical events [75,76] and as a tracker and analysis tool in evaluating the effect of public health campaigns such as tobacco control social media advocacy [77].

In this study, we set up operators to filter tweets: only those expressed in the Arabic language were included, and tweets to and from Arab states other than Saudi Arabia were excluded. The final dataset comprised 59,046 tweets (16,071 regular tweets, 2783 replies, and 40,193 retweets) and 38,066 contributors. For each tweet and contributor, variables were collected. A total of three datasets were obtained from Twitter trackers: tweet data, retweet data, and contributor data.

In collecting the newspaper data, we used Google Advanced Search to gather information on six popular Saudi national newspapers: *Al-riyadh*, *Okaz*, *Al-Madina*, *Al-Yaum*, *Al-Watan*, and *Al-Jazirah*. Across these newspapers, 518 keyword mentions were recorded. All the datasets were extracted and prepared using Excel spreadsheets (Microsoft Corp).

### Classification

The classification of data from social media, particularly Twitter, is coherent with the concept of infodemiology as described by Eysenbach [16,17] within which the study is framed/positioned.

For H1, we developed a classification to code the 2364 filtered users into four types of accounts. Individual accounts refer to any account owned by one person. Organizational accounts are those owned by a group or organization but not by media or policy organizations. Media accounts are accounts related to the media, including traditional media (programs or organizations) or news. Policy accounts are nonindividual accounts created for policy purposes or owned by political organizations.

Most of the accounts were classified in a straightforward manner as one of the authors is familiar with the Saudi environment; however, we needed to check profiles and tweets for some accounts. To validate the classification, an external observer independently classified 20% of the sample, made up of randomly chosen users from the list. The kappa [78] indicating interrater reliability was .87, indicating excellent agreement between our classification and that made by the external observer. The benchmark scale for strength of agreement proposed by Fleiss et al [79] was adopted in evaluating agreement between the study classification and that of the external observer (<40, poor; .40-.75, intermediate-good; >75, excellent).

### Preparation and Analysis

#### Tweet Dataset

From the 59,046 Twitter activities, we obtained data on 16,073 regular tweets (not retweets or replies) that mentioned any of the three Arabic keywords. Each tweet was linked to user name, time, and variables listed in Table 1.

To isolate the influential tweets, tweets with no retweets were excluded (2895 tweets). From the dataset, we extracted the account users. A retweet impression indicates the ability of a user to reach audiences that extend beyond his/her direct followers. For users with more than one tweet we selected the tweet with the highest retweet impressions (1818 tweets). By manually checking the Twitter profiles of users, we filtered out all but user accounts owned by Saudi individuals or organizations with mainly Saudi audiences. Users on the list of 1115 were classified into the four account types.

The first step in the analysis was determining the degree of influence of the groups by calculating the total impressions for each classification type. Total impressions can be an informative measure of reach, which includes all the times at which a tweet was received (including receipt by the user’s followers). Users with numerous followers can be influential in the Twitter community because of previously built influence. These users can be called Twitter influentials. To evaluate the ability of ordinary users to influence other users, we also analyzed retweet impressions, which show the total number of times a tweet was indirectly received. This is a strong measure of degree of influence, even among users with a limited number of followers.

Subsequently, a simple rank scaling measure was applied to all the users on the list. We summed the total values of all the account types. For each of the three statistics (total impressions, total retweet impressions, and retweet impressions rank scaling), we calculated the percentage of the classification types and determined the average of the retweet impressions for these classifications. This multiple test technique allows more accurate assessment in examining the study hypotheses.

#### Retweet Dataset

From the primary Twitter dataset, we obtained a list of 38,066 contributors who mentioned the keywords during the 90-day data collection period. For each contributor, we used multiple variables, including the number of retweets by user, number of impressions, and amplification multipliers. Tweetreach defines impressions as the “number of timelines that received the tweet directly from the user” and the amplification multiplier as the “rate of amplification, based on how far that contributor’s tweets spread due to the retweets and replies.” The amplification rate is calculated as follows [80]:

[(total exposure − impressions) / impressions] + 1

Users with amplification multipliers below 1.2x were filtered out in accordance with the Tweetreach evaluation [81]: “anyone with an amplification multiplier of 1.2x or higher is doing quite well at spreading conversation.” The final list included 1246 users who were coded in the classification stage. To analyze the data, we computed the total of the amplification multipliers calculated for the percentages of each classification type. We also calculated the average of the amplification multipliers for each type.

#### Contributors Dataset

Eysenbach [16], in relation to the concept of infodemiology, considers that advanced methods are required to explore the data from social networks and analyze the structures of interactions for public health. This study, rather than just identifying the presence of relationships between users on Twitter, interprets the data of contributor interactions to investigate the proposed hypotheses.

Based on total delivery ranks, we used the data on 40,193 retweets to extract data on 2665 retweets. These were all retweets over the average of deliveries, which was 2888.5. For each retweet, we identified users who retweeted a message and those who created the retweeted message. After filtering for both lists of users, 1951 unique users were classified. Type codes were used to identify 1382 users who interacted with one another.

Using R open source statistical computing and graphics software [82] we performed network analysis to explore the influence relationship among the four types of accounts. Statistics of edge interactions were calculated for each relationship, and a visualization graph was created.

#### Mention Trends

From the Twitter and newspaper data, we extracted two lists: total number of mentions in the examined newspapers and total number of mentions on Twitter. To normalize the data, all the values were divided by the maximum value in each data column, after which the data were plotted on a simple line graph. Visually, similar spikes (ie, increases in mentions) were identified in the trend mentions of the newspapers and Twitter.

## Results

### Individual and Organizational Agenda

Among the 1115 users who posted regular tweets, the number of individual accounts was considerably higher than the number of other accounts. On the basis of the total of the three measures, individual users accounted for 76.29%, 67.79%, and 96.16% of retweet impressions, total impressions, and total amplification multipliers, respectively, as shown in Table 2.

**Table 2:** Percentage totals of the three measures for the four account types.

Account types	Retweet impressions rank scale (%)	Total impressions (%)	Amplification multipliers (%)	Average (%)
Individual	76.29	67.79	96.16	80.08
Organizational	11.94	15.23	0.02	9.06
Media	9.34	11.15	0.01	6.83
Policy	2.43	5.83	3.81	4.03

As determined from the three measures, the organizational accounts dominated the media and policy accounts in terms of retweet impressions and total impressions but not in total amplification multipliers. This result indicates that policy accounts are more influential than organizational accounts based on the amplification multiplier measure.

The average of the three totals (presented in Table 2) support H1-1a and H1-1b, which project individual accounts as the dominant type and organizational accounts as more influential than media and policy accounts.

The data on contributor interactions confirm the findings derived on the basis of the three measures (Table 3). The bidirectional individual accounts dominated. As a source of influence, the individual accounts registered 80.46% influence compared with the other account types; as a target, these accounts registered 59.33% influence. Organizational accounts (17.00% and 14.74% as source and target, respectively) also exhibited higher influence than did the media and policy accounts (1.45% and 1.09% as source, respectively, and 21.27% and 4.92% as target, respectively).

**Table 3:** Contributor interactions (based on retweeting relationship). For each account type, the table illustrates how much the tweet was retweeted by other account types.

Account type	Individual (n)	Organizational (n)	Media (n)	Policy (n)	Total (n)	Percentage (%)
Individual	672	125	267	48	1112	80.46
Organizational	132	68	18	17	235	17.00
Media	8	2	9	1	20	1.45
Policy	8	5	0	2	15	1.09
Total	820	200	294	68	1382	—
Percentage (%)	59.34	14.47	21.27	4.92	—	—

### Individual Personal Agenda

In relation to the hypothesis on the power of individual accounts to influence the agendas of other Twitter users, the results do not revolve around the differentiation between individual and public agendas. Total statistical results are usually an informative indicator of mass influence, independent of the value added by the number of accounts to the total. To determine the influence of a single account, therefore, we calculated the average retweet impressions to examine the influence of both individual and public agendas. The averages indicate sustained influence of the individual agenda, which registered 72% influence in terms of total impressions and 96% in terms of the average amplification multiplier. On the basis of the contributor interactions, we assume that when an account is targeted by other account types, these accounts represent individual agendas rather than public agendas, especially when targeted individuals are not influentials or opinion leaders, as indicated by H1-2. As previously presented, the data show that the individual accounts registered 59.33% influence.

### Influence of Agenda, Not Account

As proposed, a critical requirement here is to evaluate whether influence goes beyond accounts with special characteristics. The influence of agendas, rather than the influence of accounts, was validated by two correlations. The number of deliveries and retweet impressions exhibited a very weak correlation (r=.08); the impressions and amplification multipliers showed a strong correlation (r=.01).

### Twitter and Newspaper Agenda

**Figure 4:**
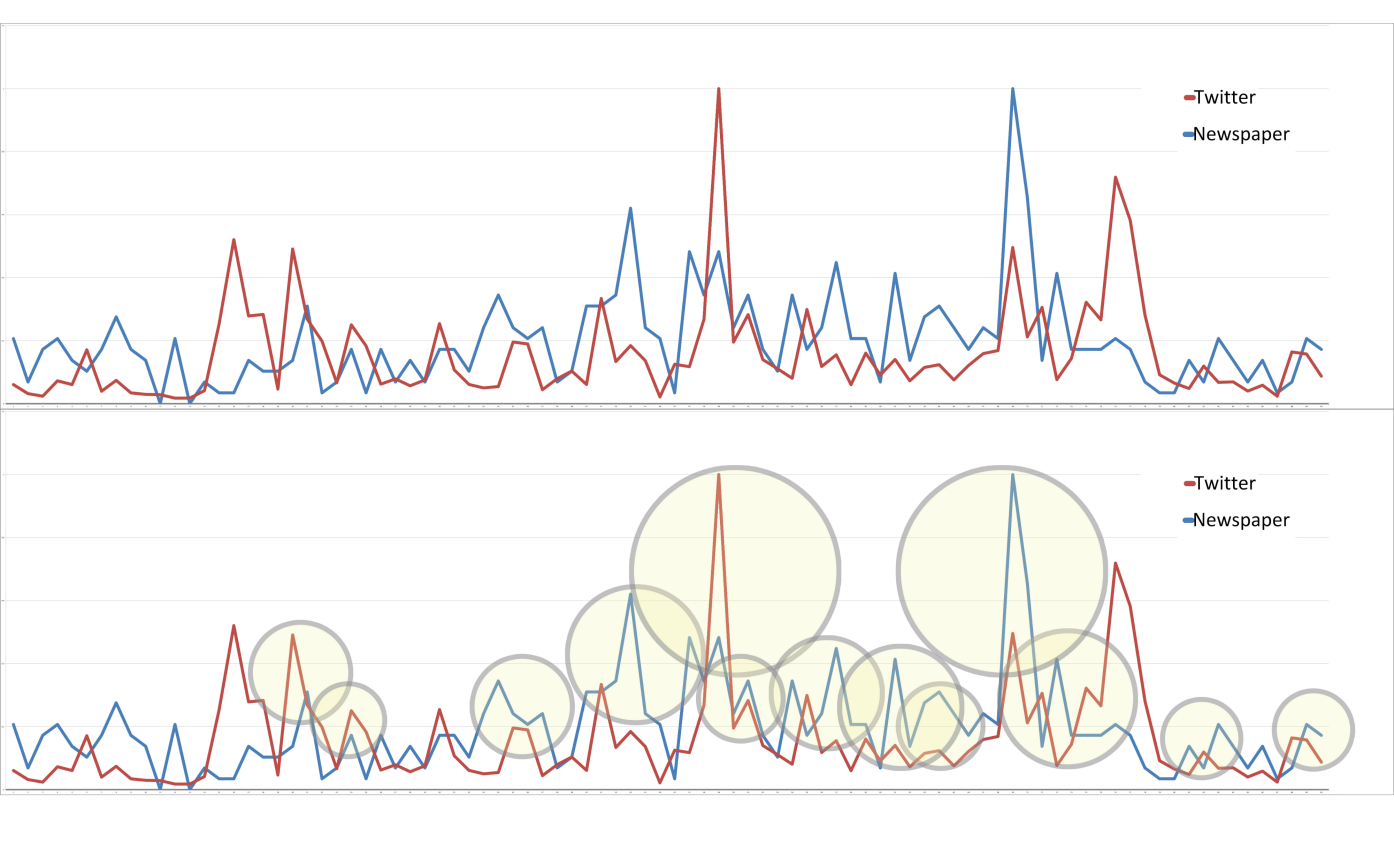
Line graph of the mention trends of Twitter and newspapers.

The line graph that represents the mention trends in Twitter and newspapers (Figure 4) shows the relationship between these two time series datasets. Broadly, we identified 13 striking similarities between the two trends.

## Discussion

### Overview

Available evidence reflects the importance of communication technologies and the Internet in daily life and social interaction [43,46]. The characteristics and features of social media facilitate the powerful effects of such platforms in terms of disseminating information, framing opinions, and mobilizing action [43,46,83].

Among the many effective features of social media, user-generated content is critical to the positioning of new channels within the agenda-setting process. Other social media functions that influence agenda-setting dynamics are the sharing of content and the selection of the type of information that users want to receive [3].

Based on a simple classification scheme, Twitter accounts can be either personal or organizational.. This study suggests that in many cases, the emergence of agenda setting among social media users (eg, Twitter users) occurs through the advocacy of individual personal agendas. On social media platforms, individuals can influence the public and organizations through their own agenda. In other words, individuals can function as independent actors in agenda setting. In a similar vein, social media platforms enable organizations and the public to influence individual perceptions and behaviors through their agendas.

From the viewpoint of health promotion, agenda setting is an effective approach to achieving best practice aims and objectives [10,11,12]. Agenda setting can hold more potential than behavioral change strategies, as confirmed by road safety research and interventions [33,84,85]. This is demonstrated through the impact of influencing social policies [84,86], persuading policy and decision makers [7], orienting media coverage [11], enhancing the advocacy process, and maximizing the diffusion of innovations [12]. In addition to influencing health policy actions, the outcomes can result in positive changes in the behaviors of individuals [87]. This indirect approach is based on the view that human behaviors are not isolated from social and community contexts [38]. The evolution of the social media age encourages health promotion practitioners and organizations to maximize the benefits of innovations arising from such developments.

This exploratory study centers on the importance of a comprehensive understanding of best practices for health promotion. It does so by suggesting a novel contribution to health promotion through the development of an adapted agenda-setting approach in the social media era and within its platforms.

### Agenda Setting in the Social Media Era

Health promotion needs a more creative approach to research and practice in using the agenda-setting function [33]. This is more challenging in the social media era where understanding such theories requires more advanced research to keep pace with the evolution of this domain.

Unlike classic agenda setting, agenda setting as a process of interaction within the social media sphere involves different actors. With evolving tools and powerful features, social media provides a unique social space characterized by rich platforms where community members can communicate and interact. Agenda setting in the context of social media remains a communication process, but it differs from traditional communication [49] in that any individual or organization can be part of the social media communication dynamic. Agenda setting via social media is a new sphere of social interaction that represents any member who desires to participate in the process. Moreover, the growth of social media use among communities enables this sphere to influence daily life.

**Figure 5:**
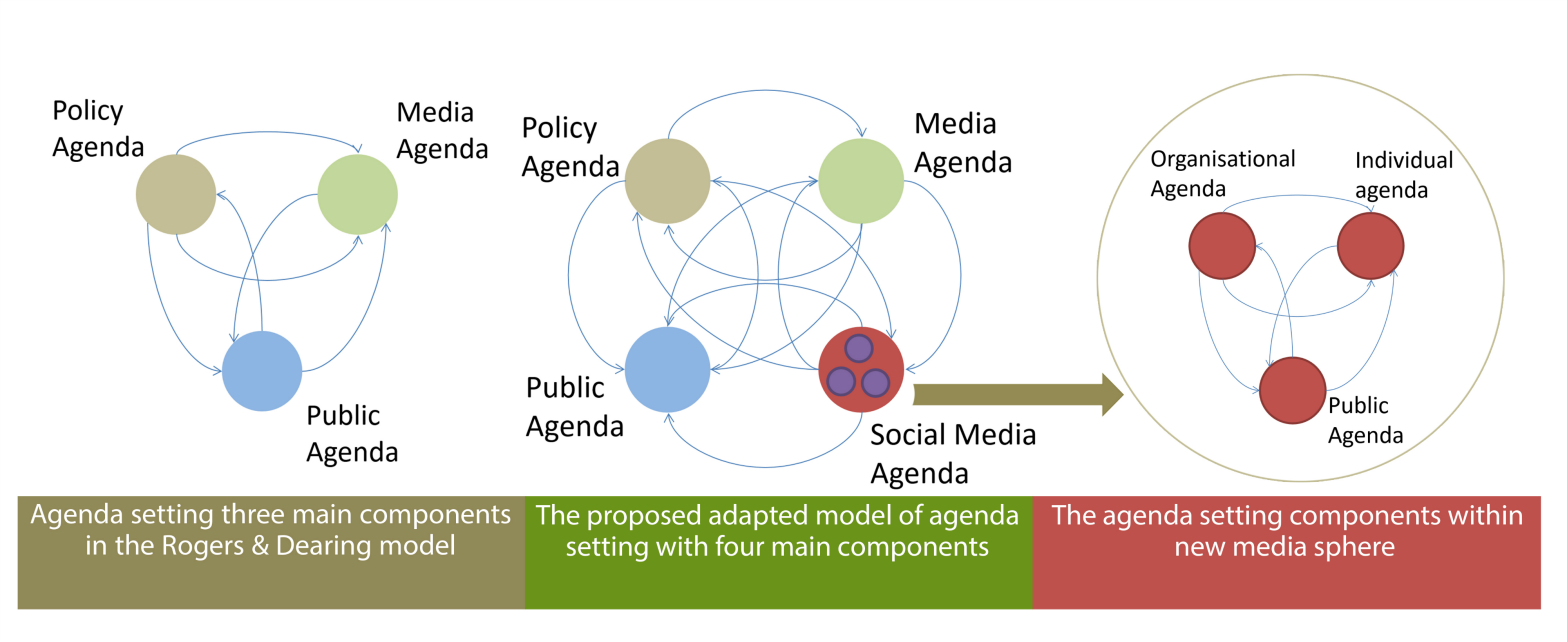
The development stages of the study proposed models built on two levels of understanding of the agenda setting process.

Given this backdrop, we suggest two levels of understanding in exploring agenda setting in the social media era. First, we propose social media as an independent agenda-formulating body within the agenda-setting process. Second, we recommend the exploration of agenda setting within social media. Figure 5 illustrates the development stages of these proposed adapted models.

### Individual and Organizational Agendas

We argue that within Twitter, agenda-setting participants can change, unlike the fixed nature of participants in classic agenda setting. This variability is attributed to the power shift towards new actors, such as individuals and organizations, as indicated in previous research [46,48,49]. We hypothesized that individuals serve as new actors in the process and that they can formulate their own personal agendas instead of adhering only to the public agenda. In addition, media and policy agendas, as part of organizational agendas, may extend to the agendas of different organizations.

The results suggest that the individual agenda dominated over the other agenda types and that organizations exhibited stronger influence than that wielded by media and policy groups. Although the data of amplification multiplier measure showed that policy accounts are more influential than organizational accounts, this influence is limited to retweets of other accounts only, which means policy accounts hold more ability to enhance the diffusion of tweets by influencing nonfollower users. In spite of this, each of the three measures used to evaluate the influence of the account types presented analogous results from various calculation methods and different variables. We believe this feature strengthens the evidence supporting the formulated hypotheses because it rectifies the limitations of one indicator or its measures.

### Individual Personal Agenda

For the individual agenda, the analysis derived different statistics which confirm that power is not restricted to the public agenda but extends to individual personal agendas. The averages of the total impressions and amplification multipliers suggest the influence of the personal agenda. The contributor interactions also support this finding, as indicated by the strong influence of the individual agenda as a target. The findings of the data analyses also highlight the influence of the public agenda, but the statistical results do not demonstrate specific differences between personal and public agendas. Agenda melding among individuals was reflected by the data on contributor interactions (672 interactions recorded).

### Influence of Agenda, Not Account

This study aimed to examine the effectiveness of agenda setting as a theory in the social media era. Therefore, a critical requirement was to consider the influence of agenda with reference to the type of account rather than account influence. The dynamics of interactions on Twitter are affected by many factors and will be/are reflected in the data [88]. A highly influential account would have generated strong bias in such an examination if it affected the values calculated on the basis of the study data. The correlation results confirm that the popularity of accounts was not an issue in the derived data values.

### Influentials Significance in Agenda-Setting Process

Although influentials or opinion leaders are beyond the scope of this work, they remain essential participants in any social communication process, including agenda setting [89]. In early theories such as the two-step flow [90] and diffusion of innovations [89], influentials have a significant impact on influencing the agenda and enhancing its diffusion. It is suggested by Kozel et al [33] that it is crucial for health promotion agenda-setting practices to develop strategies that enhance the diffusion of health promotion agendas through all agenda-setting process components. In addition to amplifying the diffusion of influence, these influentials can play a key role in sustaining the salience of specific issues, which contributes to effective agenda-setting processes [33]. The data yielded by the measures used in this study show the high impact of influentials on the agenda among users, which will increase the interrelation dynamics of influencing the agenda among different account types. Thus, using influentials is one of the strategies that can be efficient in health promotion agenda-setting practices on social media platforms. The role of influentials requires further research which is beyond the scope of the current study.

### Twitter and Newspaper Agenda

We hypothesized a relationship between newspapers and Twitter and illustrated this association via a line graph of mention trends over the platform. Nevertheless, the collected data cannot illuminate a clear direction for this relationship and do not exclude external factors that can affect the trends. This hypothesis was intended as a starting point in exploring the incorporation of the social media agenda in the classic agenda-setting process. This relationship has been studied and validated through various analysis techniques and methodologies [44,45,48,49,55,91]. Furthermore, examining causality is a crucial component of determining the direction of the relationship between new and traditional media in agenda setting [48,92].

Nonetheless, this relationship is only part of the model proposed in this work and therefore requires further examination [44,83]. Particularly interesting focal issues in this regard are the agenda-setting interaction between social media and the public agenda and that between social media and the policy agenda [44].

### Limitations

We have proposed an alternative perspective from which to understand agenda setting in the social media age. Supported by the data collected, we adopted well-defined measures that reflected a positive evaluation of the study’s hypotheses. We have applied multiple hypothesis testing methods which can support authenticity of the study as is recommended for any exploratory research [59]. Nonetheless, it is crucial in scientific research that any exploratory research must be carefully examined by confirmatory research [59].

The nature of data on social media results in limitations which can affect any infodemiology study. These include, for example, the lack of strong representation of the population and the lack of accuracy of some information provided on such platforms [17].

Furthermore, the statistical procedures and measures used require replication and/or repetition to establish a solid foundation for the implications of the results. As indicated by McCombs [45], such studies are limited by variables related to time, place, and the selection of measurement and analysis tools; a repeated examination of a model and the replication of ideas are thus critical requirements in the validation of results. Utilizing agenda setting in the field of health promotion will encounter many challenges in research and practice [93].

Many factors and variables must be considered, guided by a more in-depth understanding of the process of agenda setting in relation to its possible applications [93]. This will provide valuable strategies and themes for the successful application of agenda setting in promoting the public’s health [93].

For the public health and health promotion domain, further research may include different health topics with larger data samples and modified methods. At the organizational level, the findings suggest more dominance for health organizations in the agenda-setting process within social media. Further research related to strategies and best practices is required for such organizations to close the gap that has already been identified [53]. Moreover, such research may consider extending the infodemiology framework to development efforts addressing more topics, languages, and platforms.

### Conclusion

The results indicate that media platforms are a promising avenue that can enhance the efficacy of intervention programs. However, the effective use of such platforms will necessitate new strategies that address the limitations of traditional communication channels. More efforts towards modifying health promotion strategies and developing new approaches should be initiated. For such development, conducting research is vital to establishing a strong basis for the design, formulation, and implementation of agendas. Social media augments the effectiveness of this approach by shifting power towards reachable individual participants. In turn, agendas become more accessible and more easily used as tools or targets of influence. Finally, organizations that promote the public’s health will benefit considerably from actively participating in the agenda-setting process through the formulation of the organizational agenda.

## Appendix

Multimedia Appendix 1 Examples of intervention tweets and tweets by other users that mention the keywords road traffic accident (Arabic texts).
