# Supplementary material for: Agenda Setting for Health Promotion: Exploring an Adapted Model for the Social Media Era
Source: JMIR Public Health Surveill. 2015 Nov 25;1(2):e21. doi: 10.2196/publichealth.5014 (PMC4869225; doi:10.2196/publichealth.5014)
Supplement: Multimedia Appendix 1 [file publichealth_v1i2e21_app1.pdf]

Examples of intervention tweets and tweets by other users that mention the keywords road traffic accident (Arabic texts).

Intervention tweets:

- الصحة الجيدة مصدر رئيسي للتطور و النماء الشخصي و الاقتصادي و الاجتماعي ، و هي مهمة لجودة الحياة عموماً. #يعطيك_خيرها #وزارة_الصحة
- نسبة81% من المتوفين في مستشفيات وزارة الصحة كانت وفياتهم بسبب حوادث المرور . #يعطيك_خيرها #وزارة_الصحة
- حوادث المرور قضية صحية عامة يتولى مسؤولية الوقاية من آثارها الجهات الصحية بالإضافة لقطاعات اخرى كلاً حسب مهامه . #يعطيك_خيرها #وزارة_الصحة

Tweets by other users:

- #حياتك_شمعة_فلا_تطفئها احصائيات:توقعات بوصول الحوادث المرورية بعد 8 سنوات إلى 1,051,232
- #الحوادث_المرورية تهدر سنويًا ترليون و943 مليار ريال على مستوى العالم.
- هل تعلم ان في السعودية؟ يقتل شخص واحد كل ساعه بسبب الحوادث المرورية!!
